# Effect of purpose-directed acupuncture on the pharyngeal phase in stroke patients with dysphagia based on surface electromyography: a randomized controlled trial

**DOI:** 10.3389/fmed.2025.1565514

**Published:** 2025-05-29

**Authors:** Yan-Qiang Qin, Ying-Chun Sun, Xian-Kuan Cheng, Hai-Jiang Yao, Shao-Hong Li, Zheng-Fan Yang, Tao Zheng, Da-Peng Li, Ling-Yong Xiao

**Affiliations:** ^1^School of Rehabilitation Medicine, Treatment Center of TCM, Beijing Bo’ai Hospital, China Rehabilitation Research Center, Capital Medical University, Beijing, China; ^2^Department of Acupuncture and Moxibustion, First Teaching Hospital of Tianjin University of Traditional Chinese Medicine, National Clinical Research Center for Chinese Medicine Acupuncture and Moxibustion, Tianjin, China

**Keywords:** acupuncture, post-stroke dysphagia, purpose-directed acupuncture, randomized controlled trial, surface electromyography

## Abstract

**Introduction:**

Dysphagia following a stroke is a common complication that significantly impacts patients’ daily living abilities. Acupuncture has been reported to effectively alleviate post-stroke dysphagia and enhance patients’ quality of life. This study aims to compare the effects of purpose-directed acupuncture (PDA) schemes and conventional acupuncture schemes on post-stroke dysphagia using surface electromyography.

**Methods:**

A randomized single-blind controlled study design was employed, where eligible post-stroke dysphagia patients were randomly assigned to the treatment group and control group. Both groups received 4 weeks of treatment based on either PDA or conventional acupuncture in addition to regular dysphagia rehabilitation training. After treatment, patients’ Functional Oral Intake Scale (FOIS) scores, Standardized Swallowing Assessment (SSA) scores, and surface electromyography of swallowing muscles were assessed.

**Results:**

The study included a total of 58 subjects, with 29 in each treatment group and control group. Compared to the control group, the treatment group showed a significant increase in FOIS scores and a decrease in SSA scores. Surface electromyography results indicated that patients in the treatment group had significantly increased Aemg and Iemg values in the submental muscle group compared to the control group.

**Conclusion:**

PDA schemes may serve as a more effective treatment approach for post-stroke dysphagia, particularly showing advantages in improving submental muscle strength.

**Clinical trial registration:**

The protocol was registered in the Chinese Clinical Trial Registry (ChiCTR2100053597).

## 1 Introduction

Dysphagia is a common complication of stroke and can manifest as symptoms such as repetitive swallowing, coughing when eating or drinking, severely impacting the patient’s quality of life (1, 2). Currently, conventional rehabilitation therapies used in clinical settings only provide short-term improvement in symptoms, with overall outcomes being unsatisfactory (3). Numerous studies have found that acupuncture treatment can improve swallowing function in post-stroke dysphagia patients and animals (4–6). However, specific personalized treatment approaches tailored to address specific functional impairments, recovery stages, and rehabilitation needs are currently lacking in acupuncture treatment for dysphagia.

Purpose-directed acupuncture (PDA) is a unique acupuncture treatment plan developed by our research team through long-term clinical practice. The PDA involves an in-depth analysis of various aspects of the disease, different neurologic injury sites, individual patient differences, diverse manifestations of dysphagia, and variations in patient prognosis and outcomes. It is a differentiated and personalized acupuncture strategy aimed at the recovery of patient functional impairments. In the preliminary studies conducted by our research team, we found that the PDA treatment effectively enhances the swallowing function of stroke patients experiencing oral-phase dysphagia (7). Particularly, it demonstrates superior outcomes for managing functional impairments in the oral phase. However, whether PDA is still feasible for pharyngeal phase functional impairments remains unknown.

Here, we designed a randomized controlled study to evaluate the differences between PDA and conventional acupuncture in the treatment of post-stroke pharyngeal dysphagia using swallowing function scoring and surface electromyography. Our purpose is to identify an optimized acupuncture protocol suitable for post-stroke pharyngeal dysphagia, aiming to enhance clinical outcomes in acupuncture treatment for post-stroke dysphagia.

## 2 Materials and methods

### 2.1 Participants

Eligible post-stroke dysphagia patients who visited the Traditional Chinese Medicine Treatment Center of the Chinese Rehabilitation Research Center from Aug 2022 to July 2023 were randomly divided into a treatment group and a control group. They then received PDA treatment and conventional acupuncture treatment, respectively. This research protocol was approved by the Medical Ethics Committee of the Chinese Rehabilitation Research Center (Approval number: 2021–054-1) and registered in the Chinese Clinical Trial Registry (Registration number: ChiCTR2100053597). All participants signed informed consent forms.

Patients were enrolled if they met the following inclusion criteria: (1) Aged between 25 and 80 years old; (2) Met the diagnostic criteria for stroke (8); (3) Presented with dysphagia, scoring 2–5 on the Water Swallowing Test (9); (4) Showed pharyngeal stage symptoms of dysphagia as detected through videofluoroscopic swallowing study or endoscopic evaluation of swallowing, such as abnormal initiation of the swallowing reflex, aspiration during swallowing, inadequate displacement of the hyoid bone, presence of food retention or residue in the valleculae/pyriform sinuses/pharyngeal wall, or pharyngoesophageal reflux (10, 11); (5) Able to understand and execute simple instructions from healthcare providers, with a Mini-Mental State Examination score of ≥21 points (12); (6) Volunteered to participate in the study and signed informed consent forms.

Patients were excluded if they: (1) Had experienced stroke more than once; (2) Had experienced transient ischemic attacks; (3) Had dysphagia caused by other reasons; (4) Had severe primary diseases in the cardiovascular, hepatic, renal, hematopoietic, endocrine systems, or were psychiatric patients.

Subjects would be withdrawn and the trial terminated if they: (1) Failed to complete treatment according to the trial protocol for reasons unrelated to changes in their condition or treatment; (2) Required urgent treatment due to deterioration of their condition; (3) Experienced severe complications during the trial; (4) Stopped treatment during acupuncture therapy due to syncope or pain, making it impossible to assess efficacy; (5) Voluntarily chose to withdraw from the trial without any specific reason.

### 2.2 Study design and sample size estimation

This study is designed as a single-center, randomized, single-blind, controlled clinical trial. After reviewing the literature, it was found that the Functional Oral Intake Scale (FOIS) scores in the control group were 2.35 ± 0.62, and the expected scores in the experimental group were 3.35 ± 0.62. Using the sample size calculation software PASS 11, with a type I error probability of *α* = 0.05 and a statistical power of 90% (*β* = 0.1), it was determined that the minimum sample size per group should be 10. Considering dropout rates and result reliability, the sample size was increased to 30 cases per group.

### 2.3 Randomization and blinding

Random numbers and group assignments were generated using SAS software. The random allocation scheme was securely sealed in envelopes. Patients were enrolled and assigned to groups based on the order in which they entered, following the allocation plan inside the envelopes. Blinding procedures were applied to subjects, evaluators, and statisticians involved in the study. Basic treatments were administered by neurologists in the hospital. Therapists provided rehabilitation treatment to patients based on a designated rehabilitation training plan. Acupuncturists, according to the grouping conditions and research requirements, performed acupuncture treatment on patients in both groups. Throughout the study, apart from the acupuncturists, all other personnel were unaware of the group allocations of the patients. Before and after treatment, each patient in both groups underwent swallowing function assessments and surface electromyography examinations conducted by designated physicians. The results were recorded. Unblinding of the study occurred after the completion of the research and the processing of the collected clinical data.

### 2.4 Treatment protocol

#### 2.4.1 Conventional acupuncture treatment

During standard dysphagia rehabilitation training, acupuncture treatment was also administered. Acupoints selected included *Shuigou* (GV26), *Lianquan* (CV23), *Jinjin* (EX-HN12), *Yuye* (EX-HN13), *Fengchi* (GB20), and *Yifeng* (TE17). The posterior pharyngeal wall was also needled. Acupuncture techniques employed were as follows: (1) *Shuigou* and *Lianquan* were treated with draining method; (2) *Jinjin* and *Yuye* were pricked with a triangular acupuncture needle to induce bleeding; (3) The posterior pharyngeal wall was stimulated with a long needle insertion; (4) *Fengchi* and *Yifeng* were punctured toward the larynx, using even reinforcing and reducing techniques. Acupuncture treatment once daily with weekends off on Saturday and Sunday, for a total of 4 weeks.

#### 2.4.2 PDA treatment

The PDA treatment protocol includes the following: (1) Scalp acupuncture treatment: Bilateral motor and sensory areas are selected using acupuncture needles. After routine disinfection of the acupoints, the needles are inserted rapidly into the scalp at a 15° angle with a depth of 20–30 mm using a slight twisting technique. Electrical stimulation is applied at the lower 1/3 of the bilateral motor and sensory areas after obtaining the Qi, utilizing continuous wave mode at a frequency of 1 Hz with intensity adjusted to patient tolerance, each session lasting 30 min. (2) Neck acupuncture treatment: Bilateral *Fengchi* (GB20), bilateral *Yiming* (EX-HN14), and bilateral *Gongxue* points (1.5 cun below *Fengchi*, flat under the lower lip), are punctured directly with a depth of 20–30 mm using a slight twisting technique. Electrical stimulation is applied at the *Gongxue* points of bilateral *Fengchi* after obtaining Qi, using continuous wave mode at a frequency of 1 Hz with intensity adjusted to patient tolerance, each session lasting 30 min. For acupoints *Zhiqiang* (between hyoid bone and thyroid cartilage) and *Tunyan* (between hyoid bone and epiglottis, 0.5 inch from midline depression), direct punctures are made 3–9 mm deep. Acupoints *Lianquan*, *Jinjin*, and *Yuye* are punctured toward the base of the tongue at a depth of 20–30 mm. After rapid twisting for 15 s, needles are withdrawn from all five acupuncture points without retention. PDA treatment once daily, with weekends off on Saturday and Sunday, over a period of 4 weeks.

### 2.5 Outcome measurement

#### 2.5.1 Functional oral intake scale

FOIS indirectly determines the patient’s swallowing function based on their oral intake status (13). The scoring criteria are as follows: Score 1, Unable to take anything by mouth; Score 2, Tube dependent with minimal attempts of eating or drinking; Score 3, Tube dependent with some oral food intake of a single consistency; Score 4, Oral diet with single consistency and safe swallow; Score 5, Oral diet with multiple consistencies but requires special preparation or compensation; Score 6, Oral diet with no special preparation but specific food restrictions; Score 7, Oral diet with no restrictions.

#### 2.5.2 Standardized swallowing assessment

This evaluation consists of three steps: initial assessment, drinking 5 mL of water three times, and drinking 60 mL of water three times. A higher score indicates more severe swallowing dysfunction in patients (14).

#### 2.5.3 Surface electromyography

It involves assessing the swallowing muscle electromyographic activity in patients before treatment, immediately after the first acupuncture treatment, and at the end of the course of treatment (15). The FreeEMG300 surface electromyography system (BTS, Italy) is used for testing. The testing procedure and precautions are explained to the subjects to ensure understanding and cooperation. After cleaning the patient’s neck skin with 75% alcohol, a pair of electrodes with positive and negative poles are placed on both sides of the infrahyoid muscles along the midline of the mandible, while another pair of electrodes with positive and negative poles are placed on both sides of the thyroid cartilage to record the muscle activity of the infrahyoid muscles and the submental muscles. These muscles are superficial muscles typically involved in swallowing pharyngeal activities.

Experimental protocol: Subjects are instructed to actively swallow 3 mL of thickened food (such as thickened water or yogurt) three times. During testing, participants are advised to keep their heads still and repeat saliva swallows when the sEMG baseline signal returns to base levels for data collection.

Data collection and analysis: The EMGAnalyzer software (BTS, Italy) is used for surface electromyography signal acquisition and data analysis. The sEMG signals are filtered with a band-pass filter (50–250 Hz), undergo full-wave rectification, have a sampling frequency of 100 Hz, a common mode rejection ratio of >130 dB, gain of 1,000, and noise <1 μV. Major indices include the completion time (in seconds) of the swallowing action and time-domain analysis, including average electromyographic waveform (Aemg), integrated electromyography (Iemg), root mean square average (RMS), and maximum root mean square.

#### 2.5.4 Safety observation

It involves monitoring patient vital signs, mental status, incidence of aspiration, occurrence of secondary pneumonia, and other safety parameters. These observations are conducted before and after each treatment, with continuous monitoring for 3 days. If there are no adverse reactions, observations are then conducted weekly. It is important to observe for any adverse reactions during treatment, such as needle breakage, needle retention, fainting, bleeding, etc. If any adverse events occur, they should be promptly addressed and documented.

### 2.6 Statistical analysis

The FOIS and SSA evaluations are conducted before treatment, at the end of the treatment course, and during a follow-up after 3 months. sEMG testing is performed before treatment, immediately after the first treatment, and at the end of the treatment course. Safety observations are made before and after each treatment session. Data is collected and organized by designated personnel, and statistical analysis is carried out using SPSS 23.0 software. The chi-square test is used for categorical data, and quantitative data is presented as ± standard deviation. Comparison within groups and between groups is done using the t-test. A statistical significance level of *p* < 0.05 is considered for differences.

## 3 Results

A total of 60 cases that met the inclusion criteria were enrolled in the study. Random numbers were assigned in the order of admission, with 30 patients allocated to each group and numbered consecutively, resulting in a total of 2 groups. At the end of the study, 1 patient each from the treatment group and the control group dropped out, with a total of 58 patients being included in the final statistical analysis. Among them, there were 45 males and 13 females; aged between 27 and 80 years, with an average age of 58.78 ± 11.14 years; and a disease duration ranging from 1 to 9 months, with an average of 2.64 ± 2.23 months. Clinical data comparison between the two groups showed no statistically significant differences (*p* > 0.05), indicating comparability. General patient information can be found in Table 1.

**Table 1:** Patient general information.

Group	N	Gender		Age	Duration (month)	Classification		Site	
		Male	Female			Cerebral infarction	Cerebral hemorrhage	Hemisphere	Brainstem
PDA	29	25 (86.2%)	4 (13.8%)	56.86 ± 12.31	2.21 ± 1.92	23 (79.3%)	6 (20.7%)	19 (65.5%)	10 (34.5%)
Control	29	20 (69.0%)	9 (31.0%)	60.69 ± 9.66	3.07 ± 2.46	20 (69.0%)	9 (31.0%)	18 (62.1%)	11 (37.9%)
*p*		0.115		0.292	0.105	0.368		0.785	

### 3.1 Comparison of FOIS results between two groups

According to the findings presented in Table 2, the comparison of FOIS scores before treatment between the two groups of patients showed no statistical significance (*p* > 0.05). After treatment, both groups showed higher FOIS scores compared to before treatment, with statistical significance (*p* < 0.01). Additionally, the treatment group had higher scores compared to the control group (*p* < 0.01).

**Table 2:** FOIS score results of the two patient groups.

Group	Pre-treatment	Post-treatment	Difference before and after treatment
PDA	2.03 ± 1.24	5.24 ± 1.64	3.21 ± 1.40
Control	2.10 ± 1.29	3.00 ± 1.81	0.90 ± 0.98
*t*	0.208	4.938	7.295
*p*	0.936	0.000	0.000

### 3.2 Comparison of SSA results between two groups

As shown in Table 3, the comparison of SSA scores between the two groups of patients before treatment showed no statistical significance (*p* > 0.05). After treatment, both groups showed a decrease in SSA scores compared to before treatment, with statistical significance (*p* < 0.05). Additionally, the treatment group had lower scores compared to the control group (*p* < 0.01).

**Table 3:** SSA score results of the two patient groups.

Group	Pre-treatment	Post-treatment	Difference before and after treatment
PDA	34.93 ± 2.27	26.69 ± 4.24	8.24 ± 3.57
Control	34.90 ± 2.80	32.79 ± 3.36	2.10 ± 1.93
*t*	0.052	6.070	8.137
*p*	0.959	0.000	0.000

### 3.3 Comparison of sEMG results between two groups

**Figure 1:**
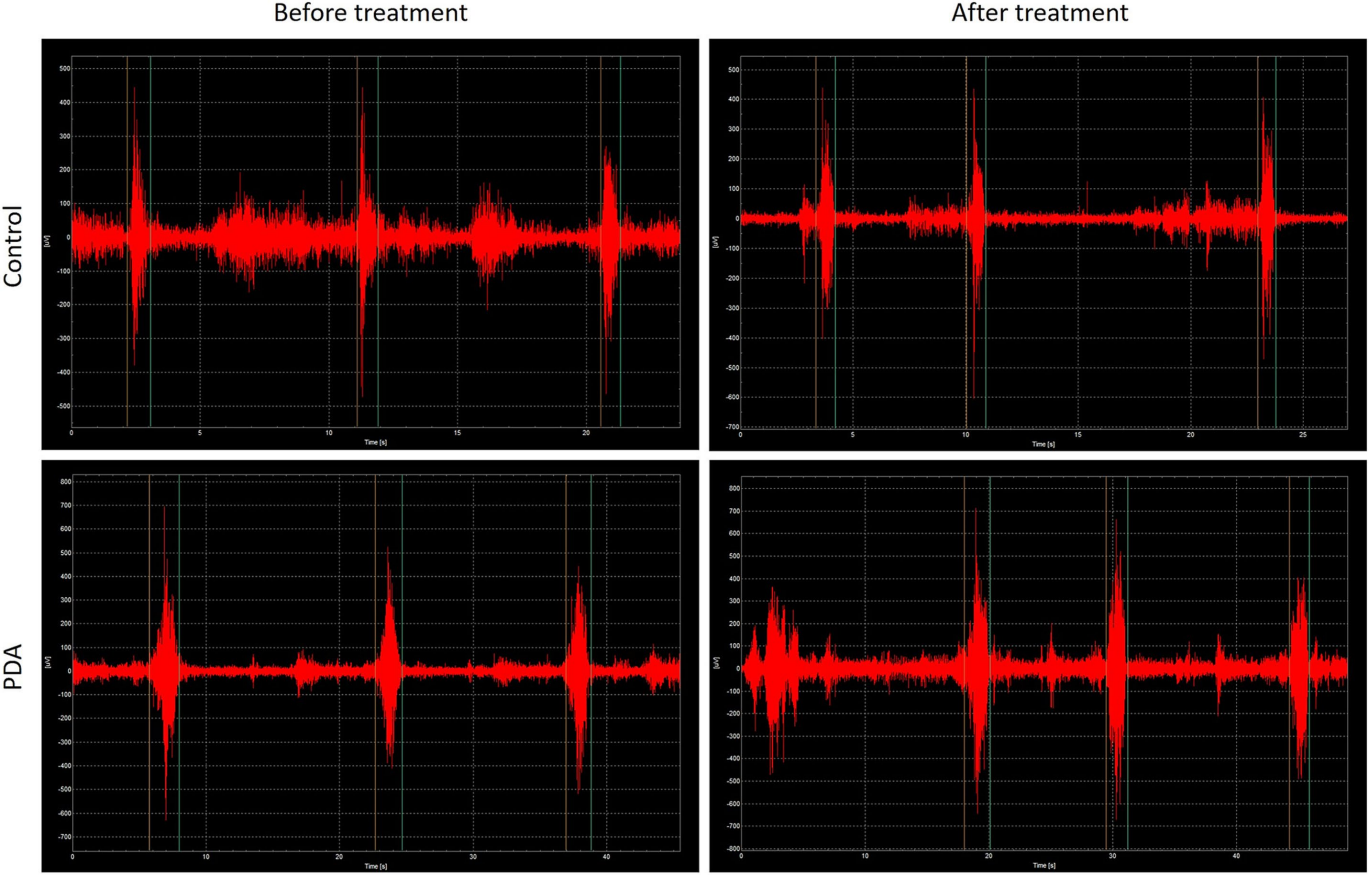
Typical sEMG diagrams of the submental muscle group in the two groups of patients.

There were no statistically significant differences in Aemg, Iemg, root mean square average values, maximum root mean square values, and swallowing timing results of the submental muscle group between the two groups of patients before treatment (Tables 4–7; Supplementary Table S1). However, after treatment, there were statistically significant differences in Iemg, root mean square average values, and maximum root mean square values of the submental muscle group between the two groups of patients (*p* < 0.05), while there were still no statistically significant differences in Aemg and swallowing timing results. There were statistically significant differences in the differences of Aemg, Iemg, root mean square average values, and maximum root mean square values of the submental muscle group before and after treatment in both groups of patients (*p* < 0.05; Tables 4–7; Supplementary Table S1). Typical sEMG diagrams of the submental muscle group in the two groups were shown in Figure 1.

**Table 4:** Aemg results of the submental muscle group in the two patient groups.

Group	Pre-treatment	Post-treatment	Difference before and after treatment
PDA	36.08 ± 15.32	58.14 ± 20.97	22.06 ± 15.32
Control	36.32 ± 15.45	47.84 ± 23.72	11.52 ± 12.49
*t*	0.600	1.752	2.872
*p*	0.952	0.085	0.006

**Table 5:** Iemg results of the submental muscle group in the two patient groups.

Group	Pre-treatment	Post-treatment	Difference before and after treatment
PDA	112.23 ± 41.53	173.01 ± 49.30	60.77 ± 41.39
Control	117.34 ± 51.89	140.25 ± 59.72	22.90 ± 21.40
*t*	0.414	2.278	4.376
*p*	0.681	0.027	0.000

**Table 6:** Root mean square average values of the submental muscle group in the two patient groups.

Group	Pre-treatment	Post-treatment	Difference before and after treatment
PDA	46.55 ± 20.93	75.08 ± 28.48	28.52 ± 27.23
Control	45.06 ± 19.25	59.74 ± 29.37	14.68 ± 15.40
*t*	0.284	2.019	2.383
*p*	0.778	0.048	0.021

**Table 7:** Maximum root mean square values of the submental muscle group in the two patient groups.

Group	Pre-treatment	Post-treatment	Difference before and after treatment
PDA	154.34 ± 53.19	240.21 ± 71.86	85.88 ± 58.43
Control	160.05 ± 82.03	196.39 ± 81.24	36.34 ± 20.39
*t*	0.315	2.176	4.311
*p*	0.754	0.034	0.000

There were no statistically significant differences in Aemg, Iemg, root mean square average values, maximum root mean square values, and swallowing timing results of the infrahyoid muscle group between the two groups of patients before treatment (Supplementary Tables S2–S6). After treatment, there were still no statistically significant differences in Aemg, Iemg, root mean square average values, maximum root mean square values, and swallowing timing results of the infrahyoid muscle group between the two groups. Only the difference in Aemg of the infrahyoid muscle group before and after treatment was statistically significant (*p* < 0.05), while there were no statistically significant differences in the other indicators (Supplementary Tables S2–S6).

### 3.4 Safety evaluation

All included patients experienced no severe adverse events.

## 4 Discussion

In this study, the effectiveness of the PDA protocol compared to conventional acupuncture in treating post-stroke dysphagia was evaluated through swallowing function scoring and sEMG analysis. The findings demonstrated a considerable advantage of the PDA protocol over conventional acupuncture in the treatment of post-stroke dysphagia. Specifically, the PDA group exhibited notable enhancements in FOIS scores and reductions in SSA scores in contrast to the conventional acupuncture group. Moreover, the sEMG analysis indicated significantly higher Aemg and Iemg values in the submental muscle group of patients receiving PDA treatment compared to those undergoing conventional acupuncture.

It is generally believed that swallowing disorders after a stroke are mainly related to damage to nerves and nuclei associated with swallowing function, such as the vagus nerve, trigeminal nerve, glossopharyngeal nerve, solitary nucleus, and nucleus ambiguous (16, 17). Acupuncture therapy has shown some clinical effectiveness in treating post-stroke swallowing disorders (18). However, for patients with oral phase swallowing disorders, especially those with dysfunctions in oral, facial, and jaw functions, accompanied by drooling, poor oral preparation, and significant post-swallowing oral residue, conventional treatments often yield unsatisfactory results (19). The PDA therapy is a treatment method that we have developed through extensive clinical practice and thorough analysis of the physiological, pathological, neuro-reflex, sensory, motor control, and swallowing compensation factors in patients with swallowing disorders. By examining the various major dysfunctions in swallowing stages, we have created this staged acupuncture treatment method. It employs a target-oriented needling approach based on precision medicine principles, specifically applied to the oral phase of post-stroke swallowing disorders.

The FOIS is a standardized assessment tool used to evaluate a patient’s ability to safely and effectively consume food and liquids by mouth (13). It consists of seven levels ranging from nothing by mouth (Level 1) to a regular diet without restrictions (Level 7), with each level representing a different degree of oral intake proficiency. On the other hand, the SSA is a clinical evaluation method that assesses various aspects of swallowing function, including oral preparatory, oral, pharyngeal, and esophageal phases. The FOIS and SSA scores provide valuable information on the individual’s swallowing abilities and help clinicians make informed decisions regarding treatment planning, diet modifications, and monitoring of swallowing function progress in patients with swallowing disorders. In the present analysis of swallowing function scores, the PDA protocol resulted in a significant improvement in FOIS scores and a decrease in SSA scores compared to conventional acupuncture. This difference in outcomes can be attributed to the unique characteristics of PDA therapy, which is more differentiated and personalized compared to traditional acupuncture, thus potentially yielding better outcomes. The personalized treatment strategies in PDA therapy may have a more direct and effective impact on the relevant muscle groups involved in swallowing, leading to improved muscle strength and coordination essential for safe and efficient swallowing post-stroke. Additionally, the increased values of Aemg and Iemg in the submental muscle group in the PDA group suggest enhanced muscle activity and coordination, further supporting the observed improvements in swallowing function scores. The specificity and precision of PDA therapy in targeting the affected muscles may explain its superiority in promoting better outcomes in the management of post-stroke dysphagia.

The sEMG is a non-invasive technique that measures the electrical activity of muscles close to the skin surface. In the evaluation of swallowing function, sEMG provides valuable insights into the coordination and strength of the muscles involved in the swallowing process (20, 21). Advancements in sEMG technology have enabled more precise and detailed analysis of muscle activity patterns during swallowing, allowing for a better understanding of the underlying mechanisms of dysphagia. Currently, sEMG has been increasingly utilized in clinical practice to assess and monitor swallowing function in patients with dysphagia, providing objective data to guide treatment planning and measure treatment outcomes. The observed increase in Aemg and Iemg values in the submental muscle group of patients receiving PDA therapy highlights the potential effectiveness of this treatment approach in improving post-stroke dysphagia. The Individualized therapy may lead to enhanced muscle activation and coordination in the submental muscle group, ultimately improving the strength and function of these muscles crucial for swallowing. This, in turn, could contribute to the overall improvement in swallowing function scores and the clinical outcomes observed in patients undergoing PDA therapy.

Several limitations need to be noted in this study. Firstly, this study was a small-sample clinical trial, and the small sample size affected the reliability of the conclusions drawn. Future research should validate the efficacy of PDA therapy in larger studies. Secondly, PDA therapy is a personalized treatment method that relies on the clinical experience and intuition of acupuncturists. The lack of standardization makes it difficult to generalize and scale up this method. Thirdly, this study only evaluated swallowing disorders in post-stroke patients and did not assess other aspects such as quality of life, depression, or anxiety. Fourthly, this study did not include a waiting treatment group, leading to a lack of a negative control group. As a result, it is difficult to determine the efficacy of acupuncture or PDA therapy for post-stroke swallowing disorders. Currently, this study can only confirm that PDA is superior to conventional acupuncture.

## 5 Conclusion

In conclusion, this study suggests that for post-stroke patients with oral phase swallowing disorders, the PDA therapy involving head, neck, tongue, and facial acupuncture points can effectively improve oral phase swallowing function and overall swallowing activity in patients. It is hoped that larger-scale randomized controlled trials will be conducted in the future to further confirm the effectiveness of PDA therapy in treating post-stroke swallowing disorders and better serve this patient population.

## Data Availability

The original contributions presented in the study are included in the article/Supplementary material, further inquiries can be directed to the corresponding authors.

## References

[1] Cohen DL Roffe C Beavan J Blackett B Fairfield CA Hamdy S. Post-stroke dysphagia: a review and design considerations for future trials. Int J Stroke. (2016) 11:399–411. doi: 10.1177/1747493016639057, PMID: 27006423

[2] Labeit B Michou E Hamdy S Trapl-Grundschober M Suntrup-Krueger S Muhle P. The assessment of dysphagia after stroke: state of the art and future directions. Lancet Neurol. (2023) 22:858–70. doi: 10.1016/S1474-4422(23)00153-9, PMID: 37596008

[3] Labeit B Michou E Trapl-Grundschober M Suntrup-Krueger S Muhle P Bath PM. Dysphagia after stroke: research advances in treatment interventions. Lancet Neurol. (2024) 23:418–28. doi: 10.1016/S1474-4422(24)00053-X, PMID: 38508837

[4] Li L Xu F Yang S Kuang P Ding H Huang M. Tongue acupuncture for the treatment of post-stroke dysphagia: a meta-analysis of randomized controlled trials. Front Neurosci. (2023) 17:1124064. doi: 10.3389/fnins.2023.1124064, PMID: 37304013 PMC10247969

[5] Yao L Liang W Du X Chen Y Huang X. Effect of acupuncture on long-term outcomes in patients with post-stroke dysphagia. NeuroRehabilitation. (2022) 51:433–41. doi: 10.3233/NRE-220113, PMID: 35871375

[6] Yao L Ye Q Liu Y Yao S Yuan S Xu Q. Electroacupuncture improves swallowing function in a post-stroke dysphagia mouse model by activating the motor cortex inputs to the nucleus tractus solitarii through the parabrachial nuclei. Nat Commun. (2023) 14:810. doi: 10.1038/s41467-023-36448-6, PMID: 36781899 PMC9925820

[7] Qin Y Yao H Sun Y Cheng X Zhou J Jing S. Study on the optimization of acupuncture in treating post-stroke dysphagia of Oral phase. Guid J Tradit Chin Med Pharm. (2021) 27:96–100. doi: 10.13862/j.cnki.cn43-1446/r.2021.08.034

[8] Chapman L Kennedy O Bradley D Harbison J. Clinical validation of in-hospital stroke diagnosis. J Stroke Cerebrovasc Dis. (2023) 32:107278. doi: 10.1016/j.jstrokecerebrovasdis.2023.107278 37549479

[9] Hongo T Yamamoto R Liu K Yaguchi T Dote H Saito R. Association between timing of speech and language therapy initiation and outcomes among post-extubation dysphagia patients: a multicenter retrospective cohort study. Crit Care. (2022) 26:98. doi: 10.1186/s13054-022-03974-6, PMID: 35395802 PMC8991938

[10] Edwards A Froude E Sharpe G. Developing competent videofluoroscopic swallowing study analysts. Curr Opin Otolaryngol Head Neck Surg. (2018) 26:162–6. doi: 10.1097/MOO.0000000000000449, PMID: 29461287

[11] Miller CK Schroeder JW Jr Langmore S. Fiberoptic endoscopic evaluation of swallowing across the age Spectrum. Am J Speech Lang Pathol. (2020) 29:967–78. doi: 10.1044/2019_AJSLP-19-00072, PMID: 32650653

[12] Carpinelli Mazzi M Iavarone A Russo G Musella C Milan G D’anna F. Mini-mental state examination: new normative values on subjects in southern Italy. Aging Clin Exp Res. (2020) 32:699–702. doi: 10.1007/s40520-019-01250-2, PMID: 31230268

[13] Crary MA Mann GD Groher ME. Initial psychometric assessment of a functional oral intake scale for dysphagia in stroke patients. Arch Phys Med Rehabil. (2005) 86:1516–20. doi: 10.1016/j.apmr.2004.11.049, PMID: 16084801

[14] Tai J Hu R Fan S Wu Y Wang T Wu J. Theta-burst transcranial magnetic stimulation for dysphagia patients during recovery stage of stroke: a randomized controlled trial. Eur J Phys Rehabil Med. (2023) 59:543–53. doi: 10.23736/S1973-9087.23.08023-1, PMID: 37737051 PMC10664766

[15] Dellavia C Rosati R Musto F Pellegrini G Begnoni G Ferrario VF. Preliminary approach for the surface electromyographical evaluation of the oral phase of swallowing. J Oral Rehabil. (2018) 45:518–25. doi: 10.1111/joor.12641, PMID: 29719051

[16] Wang Y He Y Jiang L Chen X Zou F Yin Y. Effect of transcutaneous auricular vagus nerve stimulation on post-stroke dysphagia. J Neurol. (2023) 270:995–1003. doi: 10.1007/s00415-022-11465-5, PMID: 36329182

[17] Jang SH Kim MS. Dysphagia in lateral medullary syndrome: a narrative review. Dysphagia. (2021) 36:329–38. doi: 10.1007/s00455-020-10158-3, PMID: 32654058

[18] Xie Y Wang L He J Wu T. Acupuncture for dysphagia in acute stroke. Cochrane Database Syst Rev. (2008) CD006076. doi: 10.1002/14651858.CD006076.pub2, PMID: 18646136 PMC12147868

[19] Qin Y Yao H Sun Y Cheng X Zhou J Jing S. Clinical study on the intervention of 30 stroke patients with oral-phase dysphagia by purpose-directed acupuncture protocol. Jiangsu J Trad Chinese. (2021) 53:63–6. doi: 10.19844/j.cnki.1672-397X.2021.05.023

[20] Leung KKY Fong R Zhu M Li G Chan JYK Stewart M. High-density surface electromyography for swallowing evaluation in post-radiation dysphagia. Laryngoscope. (2023) 133:2920–8. doi: 10.1002/lary.30679, PMID: 37010343

[21] Chang WH Chen MH Liu JF Chung WL Chiu LL Huang YF. Surface electromyography for evaluating the effect of aging on the coordination of swallowing muscles. Dysphagia. (2023) 38:1430–9. doi: 10.1007/s00455-023-10572-3, PMID: 37106228 PMC10471631

