# Supplementary material for: Effect of purpose-directed acupuncture on the pharyngeal phase in stroke patients with dysphagia based on surface electromyography: a randomized controlled trial
Source: Front Med (Lausanne). 2025 May 29;12:1565514. doi: 10.3389/fmed.2025.1565514 (PMC12158735; doi:10.3389/fmed.2025.1565514)
Supplement: Supplementary file 1 [file Data_Sheet_1.pdf]

Supplementary Materials

Supplemental Tables

Table S1 Swallowing timing results of the submental muscle group in the two patient groups.

Group	Pre-treatment	Post-treatment	Difference before and after treatment
PDA	1.31±0.58	0.98±0.58	0.33±0.31
Control	1.41±0.61	1.11±0.44	0.30±0.59
t	0.645	0.949	0.228
P	0.522	0.347	0.821

Table S2 Aemg results of the infrahyoid muscle group in the two patient groups.

Group	Pre-treatment	Post-treatment	Difference before and after treatment
PDA	17.60±7.49	24.36±12.17	6.77±9.80
Control	20.06±10.15	21.38±8.74	1.33±10.65
t	1.049	1.070	2.023
P	0.299	0.289	0.048

Table S3 Iemg results of the infrahyoid muscle group in the two patient groups.

Group	Pre-treatment	Post-treatment	Difference before and after treatment
PDA	46.92±20.54	64.74±45.51	17.82±40.56
Control	55.01±31.30	54.54±21.48	0.47±34.25
t	1.156	1.091	1.856
P	0.249	0.280	0.069

Table S4 Root mean square average values of the infrahyoid muscle group in the two patient groups.

Group	Pre-treatment	Post-treatment	Difference before and after treatment
PDA	21.80±9.47	32.19±22.50	10.39±18.98
Control	24.66±12.57	26.59±10.82	1.93±13.12
t	0.978	1.208	1.975
P	0.332	0.232	0.053

Table S5 Maximum root mean square values of the infrahyoid muscle group in the two patient groups.

Group	Pre-treatment	Post-treatment	Difference before and after treatment
PDA	68.62±29.19	88.84±63.04	20.22±54.32
Control	78.70±46.54	75.71±27.54	2.99±49.10
t	0.988	1.028	1.707
P	0.328	0.308	0.093

Table S6 Swallowing timing results of the infrahyoid muscle group in the two patient groups.

Group	Pre-treatment	Post-treatment	Difference before and after treatment
PDA	1.46±0.67	1.15±0.34	0.32±0.50
Control	1.62±0.59	1.28±0.44	0.34±0.64
t	0.947	1.332	1.134
P	0.348	0.188	0.894
